# Dynamic changes in the mouse hepatic lipidome following warm ischemia reperfusion injury

**DOI:** 10.1038/s41598-024-54122-9

**Published:** 2024-02-13

**Authors:** Kim H. H. Liss, Muhammad Mousa, Shria Bucha, Andrew Lutkewitte, Jeremy Allegood, L. Ashley Cowart, Brian N. Finck

**Affiliations:** 1 Department of Pediatrics, Washington University School of Medicine, St. Louis, MO USA; 2 Department of Medicine, Division of Nutritional Science and Obesity Medicine, Washington University School of Medicine, St. Louis, MO USA; 3 Washington University in St. Louis, St. Louis, MO USA; 4 Department of Biochemistry and Molecular Biology, Virginia Commonwealth University, Richmond, VA USA

**Keywords:** Lipidomics, Hepatology

## Abstract

Liver failure secondary to metabolic dysfunction-associated steatotic liver disease (MASLD) has become the most common cause for liver transplantation in many parts of the world. Moreover, the prevalence of MASLD not only increases the demand for liver transplantation, but also limits the supply of suitable donor organs because steatosis predisposes grafts to ischemia–reperfusion injury (IRI). There are currently no pharmacological interventions to limit hepatic IRI because the mechanisms by which steatosis leads to increased injury are unclear. To identify potential novel mediators of IRI, we used liquid chromatography and mass spectrometry to assess temporal changes in the hepatic lipidome in steatotic and non-steatotic livers after warm IRI in mice. Our untargeted analyses revealed distinct differences between the steatotic and non-steatotic response to IRI and highlighted dynamic changes in lipid composition with marked changes in glycerophospholipids. These findings enhance our knowledge of the lipidomic changes that occur following IRI and provide a foundation for future mechanistic studies. A better understanding of the mechanisms underlying such changes will lead to novel therapeutic strategies to combat IRI.

## Introduction

Metabolic dysfunction associated steatotic liver disease (MASLD), previously referred to as nonalcoholic fatty liver disease (NAFLD) is one of the most common causes of chronic liver disease and one of the leading indications for liver transplantation^1,2^. MASLD is characterized by accumulation of triacylglycerol resulting at least in part from elevated peripheral fatty acids from adipose tissue or dietary sources and de novo lipogenesis^3^. MASLD encompasses a spectrum of liver disease characterized by at least 5% of hepatocytes with macrovesicular steatosis with varying degrees of inflammation, cellular injury, and fibrosis^4^. The global prevalence of MASLD is estimated to be about 25–30%^4,5^. With a projected future increase in the prevalence of MASLD, the incidence of cirrhosis and hepatocellular carcinoma is also expected to increase in the next decade. As a result, MASLD related cirrhosis and cancer as an indication for liver transplantation is expected to increase further. Additionally, as steatotic livers are more susceptible to ischemia reperfusion injury (IRI), the increasing prevalence of MASLD also limits the supply of donor livers deemed suitable for liver transplantation^6,7^. IRI occurs when there is a temporary interruption in organ perfusion followed by re-establishment of blood flow. It is unavoidable in most liver-related surgeries including hepatic resections and liver transplantation. This leads to organ injury and in the case of liver transplantation, can result in primary graft non-function and early allograft dysfunction^8^. Due to increased susceptibility to IRI, the use of steatotic livers in liver transplantation has been associated with inferior patient and graft outcomes^7,9,10^.

The mechanisms that lead to increased susceptibility of steatotic livers to IRI are not well understood, and there are currently no pharmacological interventions to prevent or treat IRI. Studies in non-steatotic liver have indicated that IRI is associated with alterations in lipid metabolism^11–17^. Lipids play important physiological functions as an energy source, signaling intermediates, and building blocks for plasma membranes^18,19^. In addition to their crucial role in normal physiological processes, dysregulation of lipid metabolism and alterations in lipid composition have been recognized in pathological states such as metabolic syndrome, cancer pathophysiology, immune dysregulation, inflammatory states, and age-related diseases^20–23^. Although alterations in lipid abundance and composition have been noted after IRI, how the presence of underlying steatosis impacts dynamic changes in the hepatic lipidome is not well defined. Indeed, comprehensive hepatic lipidomic analyses comparing steatotic and non-steatotic responses to IRI have not been sufficiently addressed.

The present study was conducted under the premise that characterization of the dynamic changes in lipid composition and metabolism in steatotic and non-steatotic liver might identify novel pathophysiologic mediators of IRI. We used a well-established mouse model of warm hepatic IRI and performed unbiased, untargeted lipidomic analysis of steatotic vs. non-steatotic livers exposed to IRI at several time points after reperfusion. The relative abundance of several lipids changed dramatically after IRI and many of these were also affected by preexisting steatosis. This could facilitate identification of novel biomarkers and mechanistic targets for drug development and therapeutic intervention to ameliorate IRI.

## Results

### Hepatic steatosis exacerbates liver injury after warm partial hepatic ischemia reperfusion surgery

**Figure 1:**
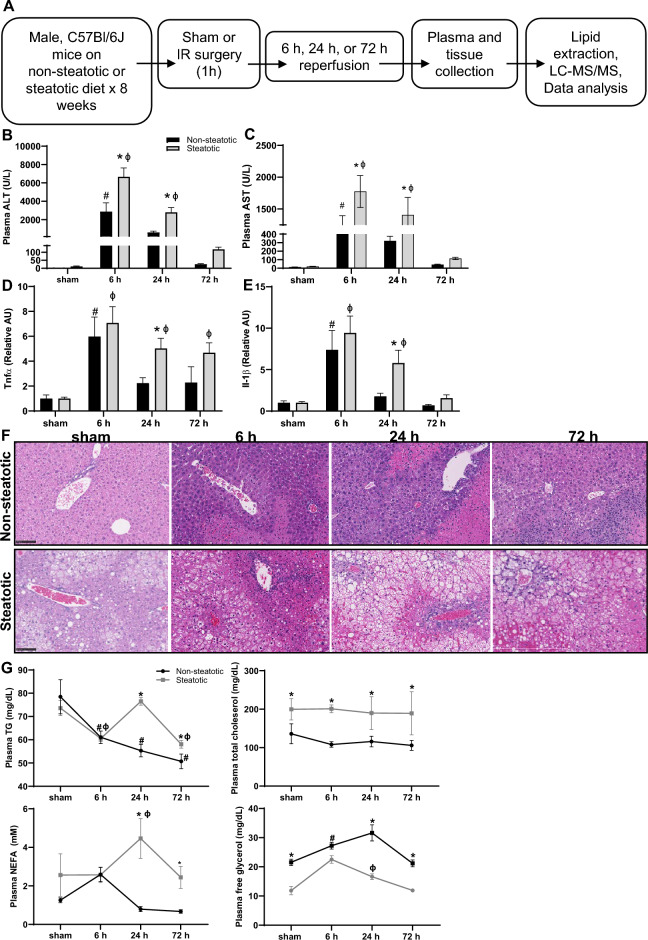
Mouse model of warm hepatic ischemia reperfusion injury, work flow, and indicators of liver injury. (**A**) Schematic of experimental design. (**B**) Plasma alanine aminotransferase (ALT) concentration following sham or IR surgery. **C.** Plasma aspartate aminotransferase (AST) concentration following sham or IR surgery. (**D, E**) Hepatic gene expression of inflammatory cytokines *Tnf* and *Il1b* following sham or IR surgery. (**F**) Liver sections stained with hematoxylin and eosin following sham or IR surgery. (**G**) Plasma lipid parameters following sham or IR surgery. Values are mean ± SEM. n = 4 per sham group, 8–10 per IR surgery group. Sham indicates sham surgery. 6 h, 24 h, 72 h indicates hours of reperfusion following IR surgery. Black bars are chow fed mice and represent non-steatotic liver. Gray bars are 42% HF fed mice and represent steatotic liver. * indicates *p* < 0.05 between non-steatotic and steatotic, # indicates *p* < 0.05 between sham non-steatotic and non-steatotic IR at specified reperfusion time point. ɸ indicates p < 0.05 between sham steatotic and steatotic IR at specified reperfusion time point. Statistical comparisons were made using ANOVA with post hoc Tukey analysis.

Starting at 6 weeks of age, male C57BL/6 J mice were fed either a standard chow diet (non-steatotic) or a diet providing 42% of its calories as fat with 0.2% cholesterol diet for 8 weeks (steatotic). Specific details regarding the two different diets are noted in Tables 1 and 2. Mice on the steatotic diet gained significantly more weight and developed fatty liver with increased intrahepatic triglyceride (TG) after 8 weeks on diet (Supplemental Fig. 1). After 8 weeks on diet, mice were subjected to either sham or IR surgery as detailed in the methods section (Fig. 1A). After surgery, mice were recovered for 6 h, 24 h, or 72 h. As expected, plasma alanine transaminase activity (ALT) and aspartate aminotransferase (AST), markers of liver injury, were elevated in all mice undergoing IR surgery compared to sham at 6 and 24 h post-surgery. ALT and AST were also significantly higher in mice with steatotic livers compared to non-steatotic livers at 6 h and 24 h post reperfusion (Fig. 1B,C). Additionally, after IR surgery, liver expression of inflammatory markers including *Tnfα* and *Il1β* was significantly increased compared to sham and significantly higher in steatotic liver compared to non-steatotic liver (Fig. 1D,E). Following IR surgery, steatotic livers had more extensive areas of hepatic necrosis compared to non-steatotic liver (Fig. 1F). These findings of increased liver injury and the temporal manifestation of this injury are consistent with our previous work in this model^14,24^.

**Table 1:** Dietary macronutrient composition.

	% kcal	
	42% high fat diet, Envigo TD.88137 (Steatotic)	Standard chow diet, PicoLab rodent diet 205,053 (Non-steatotic)
Protein	15.2	24.6
Carbohydrate	42.7	62.1
Fat	42.0	13.2

**Table 2:** Dietary fatty acid profile.

	Steatotic	Non-steatotic
% of diet		
Saturated fat	12.8	0.93
Monounsaturated fat	5.6	0.99
Polyunsaturated fat	1.0	0.33
% of total fatty acids		
Saturated fat	61.8	
Monounsaturated fat	27.3	
Polyunsaturated fat	4.7	
4:0	2.1	
6:0	1.5	
8:0	1.1	
10:0	2.6	
12:0	3.3	
14:0	10.6	
16:0	28.9	
16:1	1.5	
18:0	12.5	
18:1	20.9	
18:1 isomers	4.0	
18:2	2.3	2.19
18:2 isomers	1.3	
18:3	0.7	0.26

We then performed plasma lipid analysis (Fig. 1G). We detected significant changes in plasma triglycerides, free fatty acids (NEFA), and free glycerol between dietary groups and following IR surgery when compared to sham mice of each respective diet group. Specifically, plasma TG decreased following IR surgery at 6 h and 72 after reperfusion in both non-steatotic and steatotic diet fed mice. There were no changes to plasma total cholesterol concentrations following IR surgery in either diet groups. In steatotic mice, both NEFA and free glycerol increased in the plasma 24 h following IRI. In non-steatotic mice, there was a significant increase in plasma free glycerol following IR surgery at 6 h and a trend towards an increase in plasma NEFA at 6 h following surgery (*p* = 0.14). Thus, in addition to liver inflammation and injury, there were dynamic changes in plasma lipid content in response to IR surgery in both non-steatotic and steatotic diet fed mice.

### Hepatic lipidomic profile following IRI

To determine how IR may change hepatic lipid profiles in steatotic vs. non-steatotic livers following IR surgery, we employed untargeted LC/MS/MS. While this approach is not rigorously quantitative, it does provide a broad overview of the lipidome and changes in lipids and lipid classes can be compared between groups to gain insight into metabolic changes that occur under experimental conditions. For this, lipids were extracted from the median lobe (an ischemic lobe) from both non-steatotic and steatotic diet fed mice followed by LC–MS/MS as described in Methods. From the sham operated mice, a portion of the median lobe was also obtained for lipid extraction and analysis. Untargeted lipidomics identified 187 distinct lipid species in the following classes: diacylglycerol (DG), ceramide (Cer), cardiolipin (CL), acylcarnitine (AcCa), phosphatidylcholine (PC), phosphatidic acid (PA), phosphatidylglycerol (PG), phosphatidylinositol (PI), phosphatidylserine (PS), phosphatidylethanolamine (PE), lysophosphatidylcholine (LPC), lysophosphatidylethanolamine (LPE), lysophosphatidylserine (LPS), lysophosphatidylinositol (LPI), coenzyme Q (CoQ), hexosylceramide (HexCer), and endocannabinoids. A complete list of lipid species is delineated in Table 3.

**Table 3:** Lipid classes identified in liver following ischemia reperfusion injury.

Lipid class	Lipid species
Glycerolipids	DG(16:1_18:2), DG(16:1_18:1), DG(18:3_18:2), DG(18:2_18:2), DG(18:1_18:2), DG(18:1_18:1), DG(18:2_20:4), DG(16:0_22:6), DG(18:1_20:4), DG(18:0_20:4), DG(20:1_18:2), DG(20:1_18:1), DG(18:2_22:6), DG(22:5_18:2), DG(18:1_22:6), DG(18:1_22:5), DG(18:0_22:6), DG(22:1_18:2)
Glycerophospholipids	PA(18:0_20:4) PC(16:1_16:1), PC(16:0_16:1), PC(16:0_16:0), PC(15:0_18:2), PC(16:1_18:3), PC(14:0_20:4), PC(16:1_18:1), PC(16:0_18:2), PC(16:0_18:1), PC(18:0_16:0), PC(15:0_20:4), PC(17:1_18:2), PC(17:0_18:2), PC(17:0_18:1), PC(16:1_20:5), PC(18:3_18:2), PC(16:0_20:5), PC(18:2_18:2), PC(16:0_20:4), PC(18:1_18:2), PC(18:1_18:1), PC(18:0_18:2), PC(18:0_18:1), PC(17:0_20:4),, PC(19:0_18:2), PC(16:1_22:6), PC(18:2_20:4), PC(16:0_22:6), PC(18:1_20:4), PC(18:1_20:3), PC(18:0_20:4), PC(18:0_20:3), PC(20:0_18:2), PC(19:0_20:4), PC(18:0_22:6), PC(18:0_22:4), PC(20:0_20:4), PC(20:0_20:3), PC(20:4_22:6), PC(20:1_22:6), PC(20:0_22:6) PE(16:0_16:0), PE(16:1_18:2), PE(16:0_18:1), PE(18:0_16:0), PE(16:0p_20:4), PE(18:0p_18:2), PE(17:0_18:2), PE(18:3_18:2), PE(16:1_20:4), PE(16:0_20:5), PE(18:2_18:2), PE(16:0_20:4), PE(18:1_18:2), PE(18:1_18:1), PE(18:0_18:2), PE(18:0_18:1), PE(16:0p_22:6), PE(15:0_22:6), PE(16:0p_22:5), PE(18:0p_20:4), PE(17:0_20:4), PE(16:1_22:6), PE(18:2_20:4), PE(16:0_22:6), PE(18:1_20:4), PE(18:0_20:4), PE(20:0_18:2), PE(18:1e_22:6), PE(18:0p_22:6), PE(18:0p_22:5), PE(19:0_20:4), PE(18:3_22:6), PE(18:2_22:6), PE(18:1_22:6), PE(18:0_22:6), PE(20:0_22:6), PG(22:6_22:6) PI(16:1_18:2), PI(16:0_18:2),PI(16:0_20:5),PI(18:2_18:2), PI(16:0_20:4), PI(18:1_18:2),PI(16:0_20:3),PI(18:0_18:2), PI(18:1_18:1), PI(18:0_18:1),PI(17:0_20:4),PI(18:2_20:4), PI(16:0_22:6), PI(18:0_20:5),PI(18:1_20:4),PI(18:0_20:4), PI(18:0_20:3), PI(19:0_20:4),PI(20:4_20:4),PI(18:0_22:6), PI(18:0_22:5), PI(18:0_22:4),PI(20:0_20:4) PS(16:0_18:2), PS(16:0_20:5), PS(16:0_20:4), PS(18:0_18:2), PS(18:0_18:1), PS(16:0_22:6), PS(18:1_20:4), PS(18:0_20:5), PS(18:0_20:4), PS(20:4_20:4), PS(18:1_22:6), PS(18:0_22:6), PS(18:0_22:4), PS(20:4_22:6) LPC(16:1), LPC(16:0), LPC(17:0), LPC(18:3), LPC(18:2), LPC(18:1), LPC(18:0), LPC(19:0), LPC(20:5), LPC(20:4), LPC(20:3), LPC(20:2), LPC(20:1), LPC(22:6) LPE(16:1), LPE(16:0), LPE(18:2), LPE(18:1), LPE(18:0), LPE(22:6) LPI(16:0), LPI(18:2), LPI(18:1), LPI(18:0), LPI(20:4), LPI(20:3) LPS(18:0) CL(18:1_16:0_16:0_18:2), CL(18:2_16:1_16:1_20:3), CL(18:2_16:1_18:2_18:1), CL(18:2_18:1_16:1_18:1), CL(18:3_18:2_18:2_18:2), CL(18:2_18:2_18:2_18:2), CL(18:2_18:2_18:2_18:1), CL(18:2_18:2_18:2_22:6)
Sphingolipids	Cer(d18:1_22:0), Cer(d18:1_23:0), Cer(d18:1_24:1), Cer(d18:1_24:0), Hex1Cer(d18:1_22:0), Hex1Cer(d18:1_23:0), Hex1Cer(d18:1_24:0)
Other	AEA(16:0), AcCa(18:2), AcCa(18:0), AcCa(20:4), AcCa(20:0), AcCa(22:1), AcCa(22:0), AcCa(24:1), Co(Q8), Co(Q9), Co(Q10)

**Figure 2:**
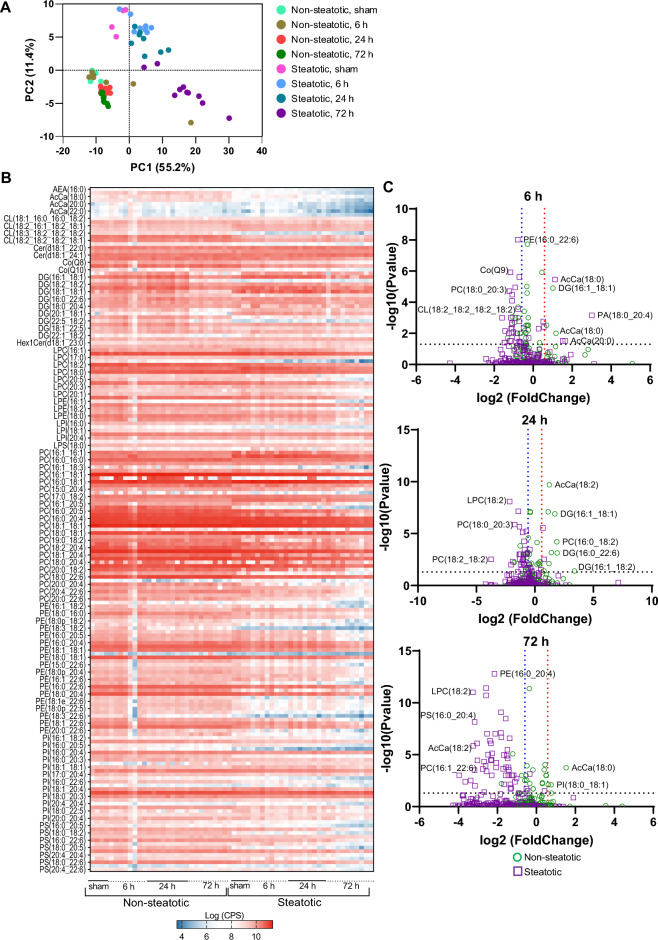
Lipidomic profile analysis. (**A**) Principal component analysis plot of the lipidomic profiles of sham operated and IR surgery groups in non-steatotic and steatotic diet fed groups. (**B**) Heat map indicating LogCPS of all identified lipid species. Every other lipid is labeled. (**C**) Volcano plots at specified reperfusion time points following IR surgery. The Log2 fold change is on the x axis, and the P value is converted to the − log10 scale is on the y axis. Fold change is relative to respective diet sham. Dashed line indicates threshold for significant *p*-value (− Log10(*p*-value) > 1.3). Non-steatotic, green circles. Steatotic, purple square. FDR adjusted *p*-values were used for multiple comparisons of lipid species.

The principal component analysis (PCA) plot highlighted differences and similarities between non-steatotic and steatotic diet fed mice and indicated that the greatest source of variation was between the two diet groups (Fig. 2A). Indeed, the non-steatotic and steatotic livers formed two distinct clusters. Within the non-steatotic group, the sham and 6 h reperfusion were similar while 24 h and 72 h reperfusion time points clustered together. In contrast, within the steatotic group, the sham, 6 h, and 24 h were similarly clustered, while the 72 h reperfusion time point formed a distinct subgroup with wide variability within its subgroup. Additionally, log transformed counts per second (cps) values of all identified lipids highlighted the changes of lipid metabolites following IR surgery and demonstrated a clear distinction between steatotic and non-steatotic liver in both sham operated animals and following IR surgery (Fig. 2B).

We then looked at individual lipid species and their fold change over their respective diet shams at all reperfusion time points (Fig. 2C). Compared to non-steatotic livers (green circles), steatotic livers (purple squares) contained more lipids that were decreased relative to sham at all reperfusion time points. While most lipid species in non-steatotic livers either remained elevated or returned to sham levels at 72 h, many lipid species were significantly decreased relative to sham in steatotic livers even 72 h after reperfusion. A complete list of significantly increased or decreased lipid species at each reperfusion time point is delineated in Supplemental Table 1.

**Figure 3:**
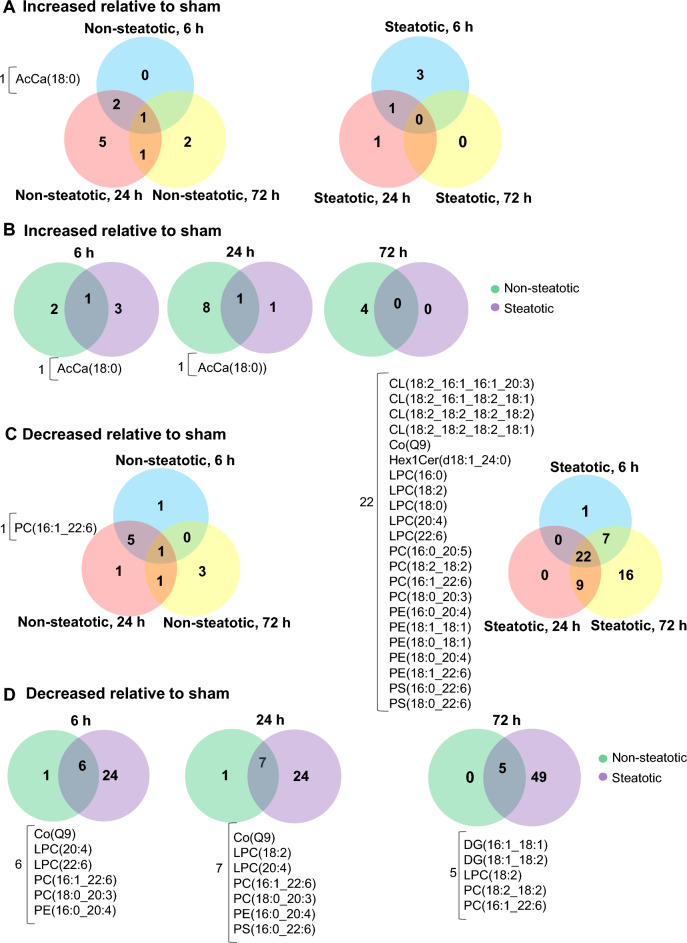
Differentially expressed lipid species. (**A**) Comparison between reperfusion time points for lipid species increased relative to sham in both non-steatotic and steatotic liver. (**B**) Comparison between non-steatotic and steatotic liver of lipid species increased relative to sham at specified reperfusion time points. (**C**) Comparison between reperfusion time points for lipid species decreased relative to sham in both non-steatotic and steatotic liver. (**D**) Comparison between non-steatotic and steatotic liver of lipid species decreased relative to sham at specified reperfusion time points. The numbers shown in overlapping areas illustrate the number of lipids commonly differentially expressed. n = 4 per sham group, 8–10 per IR surgery group. Sham indicates sham surgery. 6 h, 24 h, 72 h indicates hours of reperfusion following IR surgery. FDR adjusted *p*-values were used for multiple comparisons of lipid species.

We then compared lipids with altered relative abundance within and between diet groups (Fig. 3). Relative to sham, only a few of the identified lipids increased in both non-steatotic and steatotic liver following IRI (Fig. 3A). In non-steatotic liver, AcCa(18:0) was the only lipid that increased at all reperfusion time points. In steatotic liver, AcCa(18:0) was increased at 6 and 24 h after reperfusion (Fig. 3A). Comparison of non-steatotic and steatotic liver at individual reperfusion time points identified only one shared lipid species that increased relative to sham (Fig. 3B). We next looked at lipids that decreased relative to sham following IRI. Strikingly, in both steatotic and non-steatotic liver, the number of lipids that were significantly decreased relative to sham increased with reperfusion time, and the number of lipid species conforming to this pattern, was more pronounced in steatotic liver (Fig. 3C). In non-steatotic liver, there was only one lipid that was decreased at all reperfusion time points. In steatotic liver, there were 22 lipid species decreased at all reperfusion time points (Fig. 3C). At all reperfusion time points, there were more lipids that decreased relative to sham in steatotic liver compared to non-steatotic liver (Fig. 3D). Collectively, these data highlight the distinct differences between non-steatotic and steatotic liver and demonstrate dynamic changes in lipid profiles following IRI, which was most notable for a dramatic decrease in multiple lipid classes.

### Comparison of DG species

**Figure 4:**
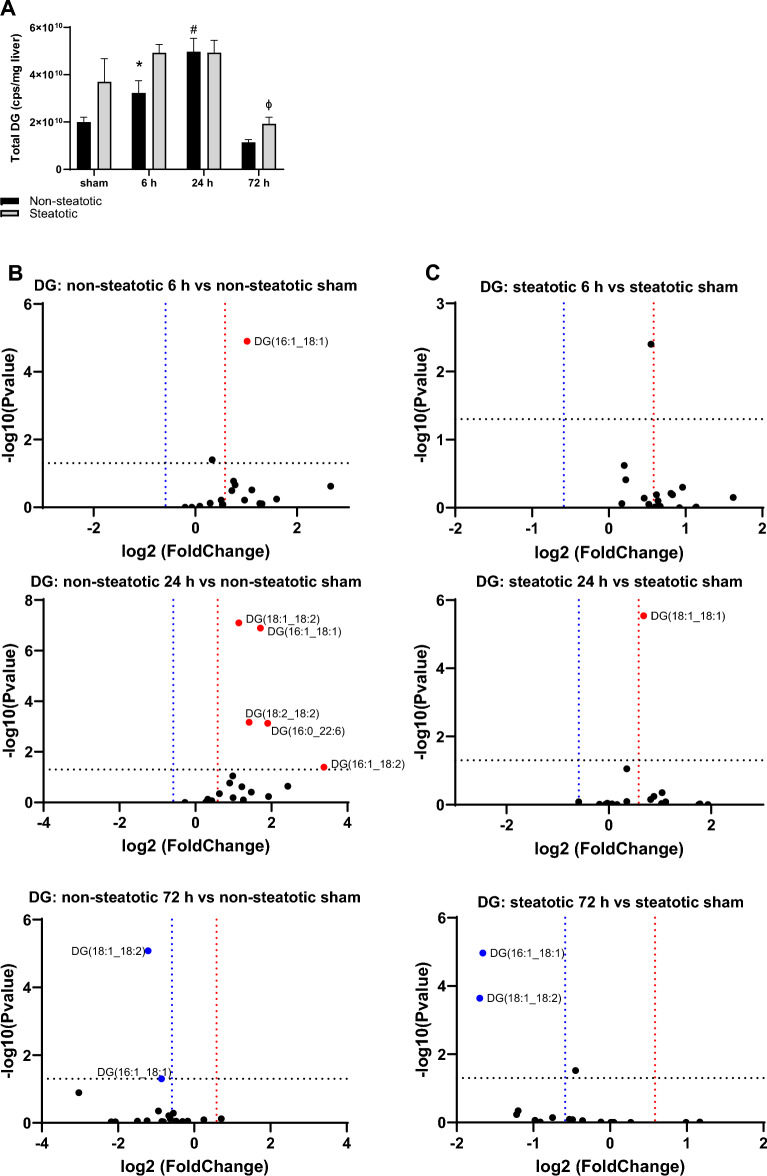
Analysis of glycerolipids. (**A**) Quantification of total diacylglycerol (DG) following sham or IR surgery. (**B**) Volcano plot of specific DG species comparing 6 h reperfusion and non-steatotic sham. (**C**) Volcano plot of specific DG species comparing 6 h reperfusion and steatotic sham. Values are mean ± SEM. n = 4 per sham group, 8–10 per IR surgery group. Sham indicates sham surgery. 6 h, 24 h, 72 h indicates hours of reperfusion following IR surgery. For volcano plots, lipids that are significantly increased relative to sham are represented in red, and those significantly decreased relative to sham are represented in blue. FDR adjusted *p*-values were used for multiple comparisons of lipid species. For bar plots, black bars are chow fed mice and represent non-steatotic liver. Gray bars are 42% HF fed mice and represent steatotic liver. Statistical comparisons for bar plots were made using ANOVA with post hoc Tukey analysis.

Total diacylglycerol (DG) increased significantly in non-steatotic liver at 24 h reperfusion compared to sham and returned to baseline at 72 h (Fig. 4A). In contrast, total DG in the steatotic liver decreased significantly at 72 h compared to steatotic sham. There was a trend towards a higher total DG content in steatotic liver (compared to non-steatotic liver) in sham operated animals and at 72 h reperfusion (*p* = 0.06 and 0.18, respectively). Total DG was significantly higher in steatotic liver compared to non-steatotic liver at 6 h. Although steatotic liver had higher total DG content, only one specific DG species was significantly increased from sham at 24 h. In contrast, in non-steatotic liver, five different DG species were significantly increased relative to chow sham at 24 h (Fig. 4B,C).

In summary, compared to sham, there was a relative increase in multiple DG species following IRI in non-steatotic liver which then decreased or returned to baseline levels at 72 h after reperfusion. However, in steatotic liver, total DG did not increase compared to sham controls. Instead, in steatotic liver, total DG decreased at 72 h relative to sham. Furthermore, when compared to steatotic liver, non-steatotic liver demonstrated more fluctuations in individual DG species with IRI compared to steatotic liver.

### Comparison of glycerophospholipids

**Figure 5:**
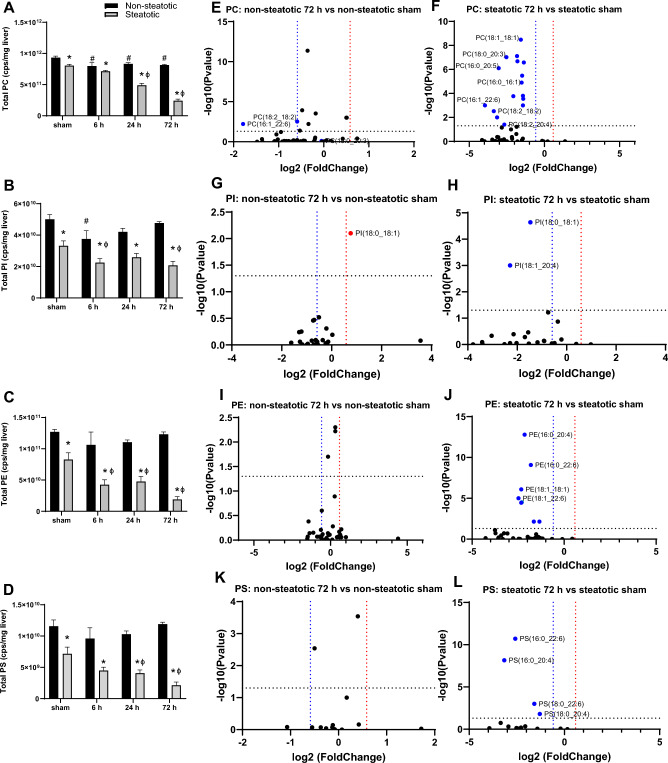
Analysis of glycerophospholipids. (**A**) Quantification of total phosphatidylcholine (PC) following sham or IR surgery. (**B**) Quantification of total phosphatidylinositol (PI) following sham or IR surgery. (**C**) Quantification of total phosphatidylethanolamine (PE) following sham or IR surgery. (**D**) Quantification of total phosphatidylserines (PS) following sham or IR surgery. (**E**) Volcano plot of specific PC species comparing 72 h reperfusion and non-steatotic sham. (**F**) Volcano plot of specific PC species comparing 72 h reperfusion and steatotic sham. (**G**) Volcano plot of specific PI species comparing non-steatotic 72 h reperfusion and sham. (**H**) Volcano plot of specific PI species comparing steatotic 72 h reperfusion and sham. (**I**) Volcano plot of specific PE species comparing 72 h reperfusion and non-steatotic sham. (**J**) Volcano plot of specific PE species comparing steatotic 72 h reperfusion and sham. (**K**) Volcano plot of specific PS species comparing non-steatotic 72 h reperfusion and sham. (**L**) Volcano plot of specific PS species comparing steatotic 72 h reperfusion and sham. Values are mean ± SEM. n = 4 per sham group, 8–10 per IR surgery group. Sham indicates sham surgery. 6 h, 24 h, 72 h indicates hours of reperfusion following IR surgery. For volcano plots, lipids that are significantly increased relative to sham are represented in red, and those significantly decreased relative to sham are represented in blue. FDR adjusted *p*-values were used for multiple comparisons of lipid species. For bar plots, black bars are chow fed mice and represent non-steatotic liver. Gray bars are 42% HF fed mice and represent steatotic liver. Statistical comparisons for bar plots were made using ANOVA with post hoc Tukey analysis.

Glycerophospholipids are important components of cellular membranes that impact membrane biophysical properties. In non-steatotic liver, total phosphatidylcholine (PC) was significantly decreased following IRI at all time points compared to non-steatotic sham (Fig. 5A). In non-steatotic liver, total phosphatidylinositol (PI) decreased at 6 h compared to sham, but returned to baseline at 24 h and 72 h (Fig. 5B). There were no significant changes in total phosphatidylethanolamine (PE) or phosphatidylserine (PS) with IRI in non-steatotic liver relative to sham (Fig. 5C,D). In the steatotic liver, total PC, PE, PI and PS were significantly decreased following IRI compared to steatotic sham. This was most dramatic at the 72 h reperfusion time point Fig. 5A–D.

There were significant differences between non-steatotic and steatotic liver in sham animals as well as all reperfusion time points for PC, PE, PI, and PS (Fig. 5A–D). The greatest difference between non-steatotic and steatotic liver was at the 72 h time point. To evaluate the 72 h reperfusion time point more closely, we looked at fold change of specific lipid species relative to the respective diet sham (Fig. 5). Strikingly, only two PC species were significantly decreased relative to sham in non-steatotic liver while more than a third of detected PC species were significantly decreased relative to sham in steatotic liver (Fig. 5E,F). PE, PI, and PS followed a similar pattern (Fig. 5G–J). Of note, one PI species (PI(18:0_18:1)) was significantly increased relative to sham in non-steatotic liver, while this same lipid was significantly decreased in steatotic liver at 72 h. None of the PS species were significantly changed relative to non-steatotic sham at 72 h reperfusion, while four PS species were significantly decreased relative to sham in steatotic liver at 72 h reperfusion (Fig. 5K,L). Together, these data indicate that there was a global disruption in glycerophospholipid content marked by a dramatic decrease in most glycerophospholipid species in steatotic liver following IRI.

### Comparison of lysoglycerophospholipids

**Figure 6:**
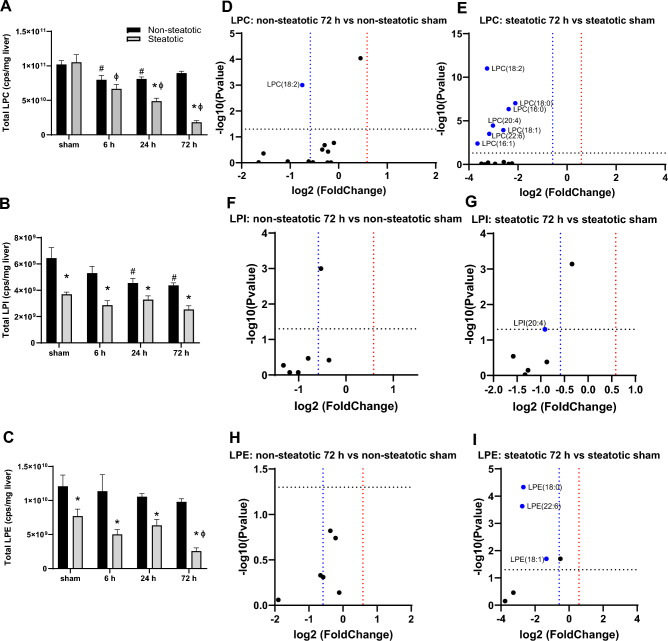
Analysis of lysophospholipids. (**A**) Quantification of total lysophosphatidylcholine (LPC) following sham or IR surgery. (**B**) Quantification of total lysophosphatidylinositol (LPI) following sham or IR surgery. (**C**) Quantification of total lysophosphatidylethanolamine (LPE) following sham or IR surgery. (**D**) Volcano plot of specific LPC species comparing non-steatotic 72 h reperfusion and sham. (**E**) Volcano plot of specific LPC species comparing steatotic 72 h reperfusion and sham. (**F**) Volcano plot of specific LPI species comparing non-steatotic 72 h reperfusion to sham. (**G**) Volcano plot of specific LPI species comparing steatotic 72 h reperfusion and sham. (**H**) Volcano plot of specific LPE species comparing non-steatotic 72 h reperfusion to sham. (**I**) Volcano plot of specific LPE species comparing steatotic 72 h reperfusion and sham. Values are mean ± SEM. n = 4 per sham group, 8–10 per IR surgery group. Sham indicates sham surgery. 6 h, 24 h, 72 h indicates hours of reperfusion following IR surgery. For volcano plots, lipids that are significantly increased relative to sham are represented in red, and those significantly decreased relative to sham are represented in blue. FDR adjusted *p*-values were used for multiple comparisons of lipid species. For bar plots, black bars are chow fed mice and represent non-steatotic liver. Gray bars are 42% HF fed mice and represent steatotic liver. Statistical comparisons for bar plots were made using ANOVA with post hoc Tukey analysis.

We next examined changes in LPC, LPE, and LPI following IRI in non-steatotic and steatotic liver (Fig. 6). The lysoglycerophospholipids followed a pattern similar to the phospholipids in response to IRI. In non-steatotic liver, total LPC and LPI decreased with IRI compared to non-steatotic sham (Fig. 6A,B), but there was no significant change in total LPE with IRI (Fig. 6C). In steatotic liver, total LPC and LPE decreased with IRI compared to steatotic sham, but there was no significant change in total LPI with IRI (Fig. 6A–C).

Comparing non-steatotic and steatotic liver, total LPE and LPI were significantly different in sham and all reperfusion time points, with higher levels in non-steatotic liver compared to steatotic liver (Fig. 6B,C). For LPC, there was a significant difference between non-steatotic and steatotic liver in sham at 24 and 72 h (Fig. 6A). We again examined specific lipid species more closely at the 72 h reperfusion time point. For both LPC and LPE, there were strikingly more individual lipid species decreased relative to sham in steatotic liver compared to non-steatotic liver (Fig. 6E,H,I). For LPI, none of the species were significantly changed in non-steatotic liver and in steatotic liver, only one LPI species were significantly decreased relative to its respective diet sham group (Fig. 6F,G).

In all, these data indicate that similar to glycerophospholipids, essentially all detected LPC and LPE species were significantly decreased in the steatotic liver following IRI. While some of the LPC and LPE species were decreased also in non-steatotic liver, this was less pronounced compared to steatotic liver.

### Comparison of mitochondrial lipids

**Figure 7:**
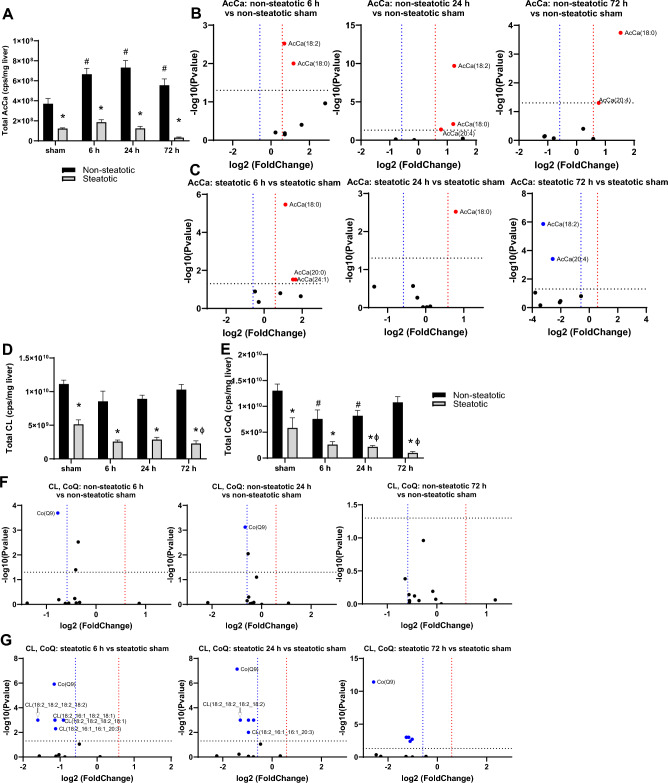
Analysis of mitochondrial lipids. (**A**) Total acylcarnitines (AcCa) following sham or IR surgery. (**B**) Volcano plot of specific AcCa species comparing non-steatotic 6 h, 24 h, and 72 h reperfusion to sham. (**C**) Volcano plot of specific AcCa species comparing steatotic 6 h, 24 h, and 72 h reperfusion to sham. (**D**) Total cardiolipin (CL) following sham or IR surgery. (**E**) Total coenzyme Q (CoQ) following sham or IR surgery. (**F**) Volcano plot of specific CL and CoQ species comparing non-steatotic 6 h, 24 h, and 72 h to CL and CoQ to sham. (**G**) Volcano plot of specific CL and CoQ lipid species comparing steatotic 6 h, 24 h, and 72 h to sham. CoQ, squares, CL, circles. Values are mean ± SEM. n = 4 per sham group, 8–10 per IR surgery group. Sham indicates sham surgery. 6 h, 24 h, 72 h indicates hours of reperfusion following IR surgery. For volcano plots, lipids that are significantly increased relative to sham are represented in red, and those significantly decreased relative to sham are represented in blue. FDR adjusted *p*-values were used for multiple comparisons of lipid species. For bar blots, black bars are chow fed mice and represent non-steatotic liver. Gray bars are 42% HF fed mice and represent steatotic liver. Statistical comparisons for bar plots were made using ANOVA with post hoc Tukey analysis.

We next examined changes in lipid species closely associated with mitochondrial function or enriched in mitochondrial membranes (Fig. 7). Acylcarnitines are synthesized to facilitate transport of fatty acyl groups across the inner mitochondrial membrane to the matrix for β-oxidation. In non-steatotic liver, total acylcarnitines (AcCa) increased with IRI compared to non-steatotic sham and AcCa(18:0) was increased at all reperfusion time points (Fig. 7A,B). In steatotic liver, total AcCa did not change following IRI relative to steatotic sham, but multiple individual AcCa species were significantly changed following IRI (Fig. 7A,C).

Cardiolipin (CL) is an abundant component of mitochondrial membranes and is exclusively localized in this compartment. In non-steatotic liver, neither total CL nor individual CL species changed significantly with IRI, compared to non-steatotic shams (Fig. 7D,F). Coenzyme Q (CoQ) transports electrons in the electron transport chain. In non-steatotic liver, total CoQ decreased with IRI at 6 h and 24 h compared to non-steatotic sham (Fig. 7E,F). In steatotic liver, there was a significant decrease in total CLs and total CoQ with IRI compared to steatotic sham (Fig. 7D,E). Additionally, in steatotic liver, multiple CLs and Co(Q9) were significantly decreased at 6, 24, and 72 h compared to steatotic sham (Fig. 7G). Compared to steatotic liver, total CLs and total CoQs were higher in non-steatotic liver in sham and all reperfusion time points following IRI (Fig. 7D,E). Collectively, these data indicate that IRI leads to a decrease in a majority of mitochondria-associated lipids in steatotic liver, but a similar decrease was not observed in non-steatotic liver.

### Comparison of sphingolipids

**Figure 8:**
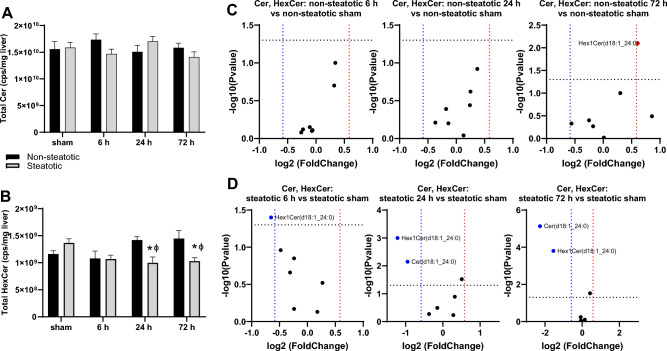
Analysis of sphingolipids. (**A**) Total ceramide (Cer) following sham or IR surgery. (**B**) Total hexosylceramide (HexCer) following sham or IR surgery. (**C**) Volcano plot of specific Cer (circles) and HexCer (squares) species comparing non-steatotic 6 h, 24 h, and 72 h to sham. (**D**) Volcano plot of specific Cer (circles) and HexCer (squares) species comparing steatotic 6 h, 24 h, and 72 h to sham. Values are mean ± SEM. n = 4 per sham group, 8–10 per IR surgery group. Sham indicates sham surgery. 6 h, 24 h, 72 h indicates hours of reperfusion following IR surgery. For volcano plots, lipids that are significantly increased relative to sham are represented in red, and those significantly decreased relative to sham are represented in blue. FDR adjusted *p*-values were used for multiple comparisons of lipid species. For bar blots, black bars are chow fed mice and represent non-steatotic liver. Gray bars are 42% HF fed mice and represent steatotic liver. Statistical comparisons for bar plots were made using ANOVA with post hoc Tukey analysis.

Sphingolipids play a variety of important roles in regulating intracellular signaling cascade and membrane dynamics and can also be classified into subtypes, including ceramides and hexosylceramides. Total ceramide content did not change with IRI at any time point in both non-steatotic and steatotic liver (Fig. 8A). In non-steatotic liver there was a trend towards increased total HexCer content at 24 h and 72 h relative to sham, but this did not reach statistical significance (*p* = 0.37 and *p* = 0.11, respectively) (Fig. 8B). In steatotic liver, there was a significant decrease in total HexCer at 24 h post reperfusion compared to steatotic sham (Fig. 8B). We did not detect any significant difference in total ceramide content between non-steatotic and steatotic liver in sham operated animals or at any reperfusion time point (Fig. 8A). Non-steatotic liver had a significantly higher total HexCer content than steatotic liver at 24 h and 72 h post reperfusion (Fig. 8B).

We then looked at individual ceramide species. In non-steatotic liver, Hex1Cer(d18:1_24:0) was the only species that significantly changed relative to non-steatotic sham following IRI at any reperfusion time point (Fig. 8C). In steatotic liver, Cer(d18:1_24:0) and HexCer(18:1_24:0) were significantly decreased compared to steatotic sham (Fig. 8D).

### Correlation between lipid species and plasma alanine transaminase

**Figure 9:**
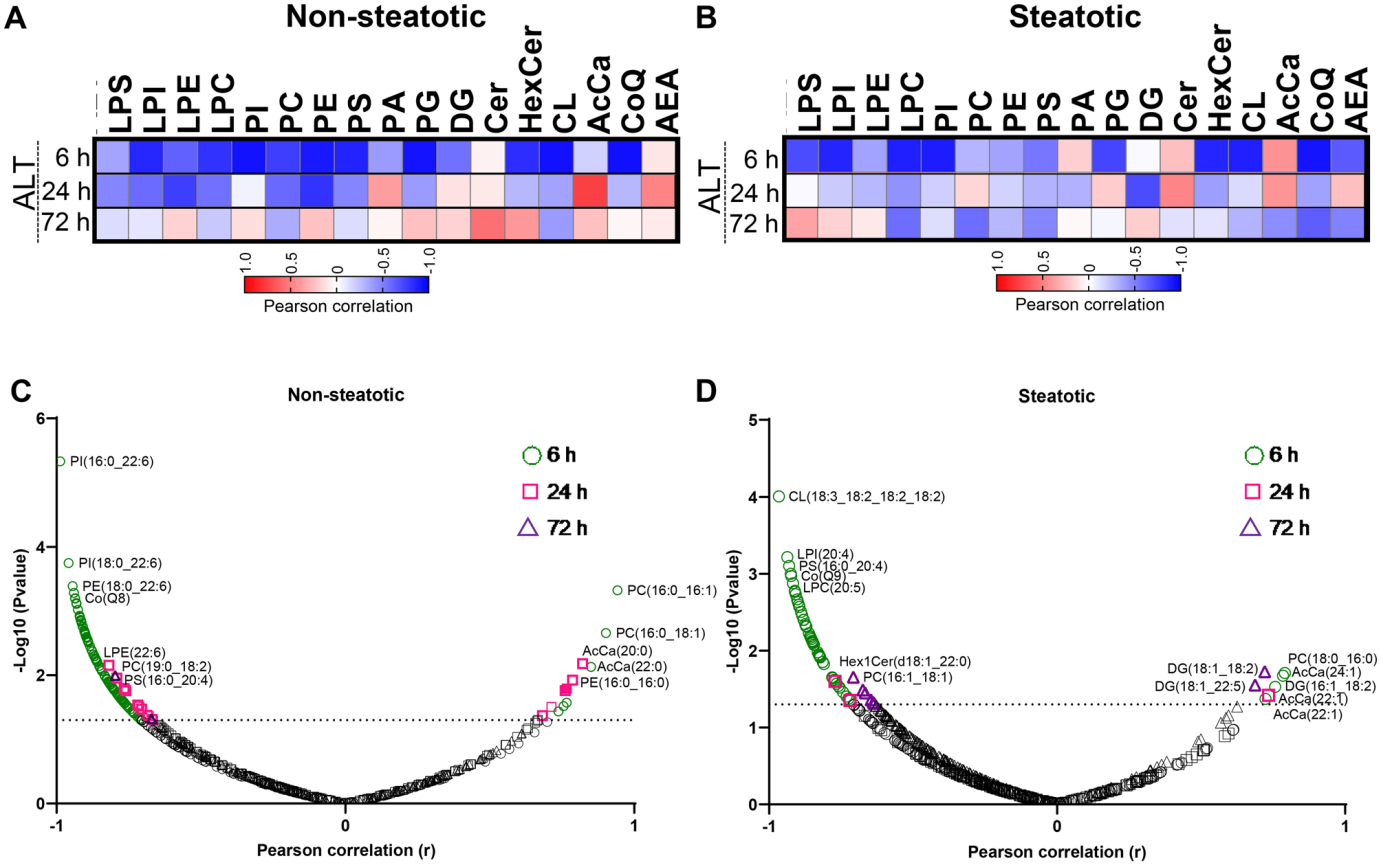
Pearson correlation matrix. (**A**) Correlation between plasma ALT and lipid class in non-steatotic diet fed mice at 6 h, 24 h, and 72 h post reperfusion. (**B**) Correlation between plasma ALT and lipid class in steatotic diet fed mice at 6 h, 24 h, and 72 h post reperfusion. Red indicates positive correlation. Blue indicates negative correlation. (**C**) Pearson correlation between plasma ALT and specific lipid species in non-steatotic diet fed mice at all reperfusion time points. (**D**) Pearson correlation between plasma ALT and specific lipid species in steatotic diet fed mice at all reperfusion time points. Dashed line on volcano plots indicate threshold for significant *p*-value (− Log10(*p*-value) > 1.3) plotted on y axis. Pearson correlation coefficient plotted on x axis.

As plasma ALT is the most common marker of liver injury, we next looked for correlations between ALT values and specific lipid species (Fig. 9). Pearson correlation coefficients were calculated using all individual ALT and cps values at each reperfusion time point. In non-steatotic liver, at 6 h reperfusion, ALT was significantly negatively correlated with LPI, LPC, PI, PC, PE, PS, PG, HexCer, CL, and CoQ. ALT was not significantly positively correlated with any lipid class measured at 6 h and 72 h. At 24 h, ALT was significantly negatively correlated with LPE and PE and positively correlated with AcCa (Fig. 9A). In steatotic liver, at 6 h reperfusion, total LPI, LPC, PI, PG, HexCer, CL, and CoQ were significantly negatively correlated with plasma ALT. At 24 h reperfusion, none of the lipid classes were significantly associated with plasma ALT. At 72 reperfusion, only total CoQ was significantly negatively correlated with plasma ALT.

As noted above, we often detected significant changes in individual lipid species even when no changes were detected from the total class after IRI. Thus, we examined each individual lipid species for correlation with plasma ALT in both non-steatotic and steatotic diet fed mice (Fig. 9C,D). In non-steatotic liver, 109 individual lipid species were significantly correlated with plasma ALT at 6 h, 22 lipid species at 24 h, and two lipid species at 72 h. In steatotic liver, 55 lipid species were significantly correlated with plasma ALT at 6 h, three at 24 h, and nine at 72 h reperfusion (Fig. 9C, Supplemental Table 2).

We then examined how the correlated lipid species changed with IRI. In non-steatotic liver, at 6 h reperfusion, none of the lipid species positively correlated with plasma ALT, were significantly increased or decreased relative to non-steatotic sham. Of the lipid species negatively correlated with plasma ALT at 6 h, six were significantly decreased and one was significantly increased (AcCa(18:2)) relative to non-steatotic sham. In non-steatotic liver, of the lipids positively correlated with plasma ALT at 24 h, AcCa(18:2) was significantly increased relative to non-steatotic sham and none were significantly decreased relative to non-steatotic sham. Of the lipids negatively correlated with plasma ALT at 24 h, none were significantly increased or decreased relative to non-steatotic sham. At 72 h reperfusion, of the two lipid species negatively correlated with plasma ALT, only PC(18:2_18:2) was significantly changed (decreased) relative to sham in non-steatotic liver.

In steatotic liver, of the lipid species that were positively correlated with plasma ALT, only AcCa(24:1) was significantly increased and none were significantly decreased at 6 h. In contrast, of the lipids that were negatively correlated with plasma ALT at 6 h, many were significantly decreased relative to steatotic sham. At 24 h reperfusion, of the lipids negatively correlated with plasma ALT in steatotic liver, only PC(16:0_20:5) was also significantly decreased relative to steatotic sham. Interestingly, of the 54 lipids that were significantly decreased following IRI in steatotic liver at 72 h, only two were correlated with plasma ALT. Of the lipid species negatively correlated with plasma ALT at 72 h, only PC(16:1_18:1) was significantly decreased at 72 h reperfusion compared to steatotic sham. Of the lipid species positively correlated with plasma ALT in steatotic liver at 72 h, only DG(18:1_18:2) was significantly changed relative to sham.

Collectively, these data indicate that while many lipid species fluctuated following IRI, a relatively small percentage were correlated with plasma ALT. Of these correlations, a general pattern was noted whereby phospholipids were negatively correlated with plasma ALT and in general tended to decrease following IRI. This finding was more pronounced in steatotic liver and persisted at the 72 h reperfusion time point.

## Discussion

Ischemia reperfusion injury is largely unavoidable in most liver-related surgeries, and the presence of steatosis exacerbates injury. There are no specific biomarkers used for diagnosing or prognosticating hepatic IRI, and there are no pharmacological therapies available to prevent or treat IRI. While it is generally thought that accumulation of toxic lipid species is linked to hepatic inflammation and fibrosis^25,26^, how specific lipids impact IRI is less well understood. In this study, we used an unbiased, untargeted approach to systematically evaluate changes in lipid composition following IRI in both non-steatotic and steatotic liver in mice.

Broad analysis indicated that steatotic and non-steatotic liver have distinct changes to intrahepatic lipid profiles following IRI. This highlights the need to perform studies in both non-steatotic and steatotic liver, as a positive response to intervention in one may not be applicable to the other. Interestingly, even when content of a given total lipid class did not change significantly with IRI, we noted fluctuations in specific lipids within that class. These changes in composition may reflect fatty acid availability or substrate preferences. This will require further investigation as previous studies have indicated that specific fatty acids may influence IRI outcomes^27,28^. Additionally, we noted that there were very few lipids that increased relative to sham following IRI in either non-steatotic or steatotic liver. Of note, PA(18:0_20:4), the only PA detected in our study, was increased at 6 h reperfusion in steatotic liver, but unchanged in all time points in non-steatotic liver (Supplemental Fig. 2). PA has mitogenic effects and may play a role in liver regeneration^14,29–33^. The increase in PA in steatotic liver may be a reflection of increased liver injury, and thus a need to initiate regeneration following IRI.

**Figure 10:**
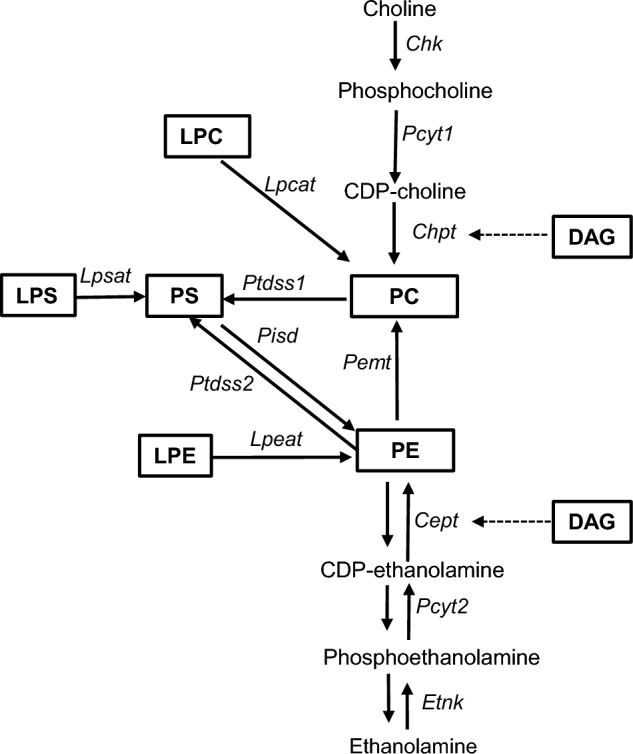
Pathway of select glycerophospholipid and lysoglycerophospholipid metabolites. PC, phosphatidylcholine; PS, phosphatidylserine; PE, phosphatidylethanolamine; LPC, lysophosphatidylcholine; LPS, lysophosphatidylserine LPE, lysophosphatidylethanolamine; DAG, diacylglycerol; Italicized abbreviations represent enzymes; Chk, choline kinase; Pcty1, CTP: phosphocholinecytidyltransferase; Chpt, cholinephosphotransferase; Etnk, ethanolamine kinase; Pemt, PE methyltransferase; Cept, ethanolaminephosphotransferase; Pcyt2, CTP: phosphoethanolaminecytidyltransferase; Ptdss, phosphatidylserine synthase; Pisd, phosphatidylserine decarboxylase; Lpcat, lysophosphatidylcholine acyltransferase; Lpsat, lysophosphatidylcholine acyltransferase;

With the exception of PA, total phospholipids and glycerophospholipids, specifically total PC, PE, PI, PS, LPC, and LPE, all relatively decreased with IRI in steatotic liver following IRI suggestive of generalized downregulation of the Kennedy Pathway with IRI (Fig. 10). Given the striking decrease in PC and PE in steatotic liver compared to non-steatotic liver, it is possible that the relative increase in DAG we observed in non-steatotic liver may be feeding into the Kennedy Pathway to compensate (Fig. 10). A similar increase in DAG was not seen in steatotic liver. We postulate that in the setting of pre-existing steatosis, there may be less metabolic flexibility. Furthermore, we found multiple PC and PE lipid species to be negatively correlated with plasma ALT, suggesting that decreased phospholipid content is associated with increased liver injury. Phosphatidylcholine has an essential role in the assembly of cell membranes, lipoproteins, lipid droplets and bile synthesis^34^. Aberrant phosphatidylcholine metabolism has been linked to MASLD and liver failure^35–37^, cardiovascular disease, myocardial ischemia reperfusion injury^38,39^, and Alzheimer’s disease^40,41^. Furthermore, liver regeneration following partial hepatectomy is influenced by the PC:PE ratio^42^ and similar to our findings, in the murine acetaminophen model, almost all PC species decreased following acetaminophen treatment^43^. A recent multiomics study by Hall et al.^44^ suggests that hepatocyte proliferation is associated with increased de novo synthesis of PC. The role of phospholipid metabolism has not been specifically investigated in hepatic IRI, but PC administration has been shown to decrease intestinal IRI^45^ and brain IRI^46^. While Zazueta et al.^47^ found CDP-choline to ameliorate IRI in non-steatotic liver, they did not evaluate effects in steatotic liver and did not measure lipid levels. Given the important role of PC as the predominant phospholipid in cell membranes, the relative decrease in PCs following IRI compared to non-steatotic livers may contribute to slower resolution of and recovery from IRI in steatotic liver. Thus, future studies will need to evaluate PC content as prognostic markers and the role of PC supplementation in hepatic IRI.

Interestingly, we found that AcCa was positively correlated with plasma ALT in both non-steatotic and steatotic liver. Changes in AcCa likely result from alterations in fatty acid oxidation seen in ischemia reperfusion injury^48,49^. To our knowledge, there have not been previous studies evaluating a relationship between AcCa and hepatic IRI. Previous work has reported beneficial effects of L-carnitine in rat hepatic IRI^50^. Liepinsh et al.^51^ previously found decreasing acylcarnitine content was associated with decreased myocardial IRI. However, future studies evaluating the effects of AcCa on hepatic IRI are needed.

In addition to our novel findings, our study also reports changes that are consistent with previous studies. For example, we found cardiolipin, a phospholipid exclusive to the mitochondria, to be significantly decreased relative to sham in steatotic liver following IRI and negatively correlated with plasma ALT. Multiple groups in cardiac^52^ and hepatic^15^ IRI have reported similar findings suggestive of mitochondrial dysfunction secondary to IRI. We also noted decreased CoQ following IRI and previous studies have noted alleviation of IRI with administration of CoQ10^53–55^. Of note, previous studies^13,56–58^ have noted changes in ceramide content following IRI, but we did not detect such alterations. This may be due to differences in study design and methodology.

There are several limitations to our study. Importantly, this study is descriptive in nature and untargeted lipidomics cannot provide absolute quantification. While not intended to define the mechanisms by which alterations in lipid composition occur, it lays the foundation for design of future mechanistic studies. Further studies including transcriptomic analysis are needed to fully evaluate how altered lipidomics may contribute to IRI and how such changes may be exploited for therapeutic potential. Second, we only utilized one murine model NAFLD. However, our model is most relevant as genetic models of obesity and the methionine choline deficient diet are less physiologically applicable. Additionally, the MASLD produced by the 42% HF diet creates a reliable, reproducible model of steatosis and inflammation without significant fibrosis. Third, female mice were not included in this study, and there may be sex-specific responses to IRI. Future targeted lipidomic studies should include both male and female mice to explore such differences. Finally, in this study, we did not perform comprehensive plasma lipidomics. However, our primary aim was to characterize hepatic lipidomic changes as this is the site of primary injury. Plasma lipidomics would reflect more systemic changes or changes to other organs such as adipose tissue following IRI. As noted by others, adipose tissue dysfunction plays a key role in insulin resistance and may play a role in hepatic lipidome remodeling in the setting of MASLD^59,60^. Recent studies have also highlighted key differences in serum lipidomic profiles of healthy and MASLD patients noting depletion of several phospholipid classes^61^. Thus, in the future, it would be important to further delineate changes in plasma lipids as this would allow for study of systemic effects of IRI and help identify novel non-invasive biomarkers.

It is thought that steatotic livers are more susceptible to IRI due to underlying mitochondrial dysfunction, ER stress, and microcirculatory impairment that exaggerates liver injury and cell death following IRI. Hence, most studies have focused on interventions that would target these pathways. However, dysregulated lipid metabolism and alterations in lipid composition have been shown to contribute to various disease states. In our present study, utilizing unbiased, comprehensive lipidomic analysis, we have illustrated that there are distinct and dynamic changes to lipid profiles following IRI in non-steatotic and steatotic liver. Specifically targeting lipid metabolism represents a novel therapeutic approach. Our findings expand our knowledge of the lipidomic changes that occur and provide valuable insight regarding biomarker identification and therapeutic strategies.

## Methods

### Animals

Male C57BL/6 J mice were purchased from Jackson Laboratory (Bar Harbor, ME). At 6 weeks old, mice were continued on standard chow diet (PicoLab Rodent diet 205,053) or transitioned to a diet with high fat (42% calories), sucrose (34% calories), and cholesterol (0.2% w/w) (42% HF; TD 88,137, Envigo, Indianapolis, IN) (Tables 1 and 2). Mice were maintained on diet for 8 weeks prior to surgery. All animal studies were approved by the Institutional Animal Use and Care Committee of Washington University School of Medicine and comply with the *Guide for the Care and Use of Laboratory Animals* as outlined by the National Academy of Sciences. The study is reported in accordance with ARRIVE guidelines.

### Hepatic ischemia reperfusion surgery

Hepatic ischemia was induced using a 70% ischemia model as previously described^14,24,62^. Briefly, mice were anesthetized using isoflurane inhalation. Midline laparotomy was performed followed by cross-clamping of the hepatic artery, portal vein, and bile duct distal to the branch point to the right lateral lobe to induce ischemia to the median and left lobes. The atraumatic clamp was released after 1 hour followed by 6, 24, and 72 h reperfusion. Mice undergoing sham surgery underwent midline laparotomy with vascular clamping and were maintained under isoflurane anesthesia for 1 h. At the predetermined reperfusion time point, mice were euthanized using carbon dioxide and plasma and liver samples were collected for analysis.

### Plasma parameters

Plasma alanine aminotransferase (ALT) and aspartate transaminase (AST) were measured using commercially available colorimetric kinetic assays (Teco Diagnostics, Anaheim, CA) according to manufacturer’s instructions. Plasma nonesterified fatty acids, total cholesterol, and triglycerides were measured using commercially available colorimetric kits (Wako Diagnostics, Mountain View, CA; and Thermo Fisher Scientific) according to the manufacturer’s instructions.

### Liver triglyceride assay

A portion of the median lobe was obtained at time of sacrifice. Liver samples were then homogenized in saline followed by the addition of 1% sodium deoxycholate to solubilize lipids. Hepatic triglyceride content was measured using a commercially available enzymatic assay (Thermo Fisher Scientific, Waltham MA).

### Histology

A portion of the left lateral lobe was harvested at the time of sacrifice and placed in 10% neutral buffered formalin followed by 70% ethanol. The tissues were then embedded in paraffin, sectioned, and stained with hematoxylin–eosin (H&E) stain.

### Untargeted LC/MS/MS lipidomics

Internal standards were purchased from Avanti Polar Lipids (Alabaster, AL) as their premixed SPLASH LIPIDOMIX mass spec standard. Standards included 15:0–18:1(d7) PC, 15:0–18:1(d7) PE, 15:0–18:1 (d7) PS, 15:0–18:1 (d7) PG, 15:0–18:1 (d7) PI, 15:0–18:1 (d7) PA, 18:1 (d7) LPC, 18:1 (d7) LPE, 18:1(d7) cholesterol ester, 18:1(d7) MAG, 15:0–18:1(d7) DAG, 15:0–a8:1(d7)–15:0 TG, 18:1(d9) SM, and cholesterol (d7). For LC–MS/MS analyses, a Thermo Scientific Q Exactive HF Hybrid Quadrupole-Orbitrap Mass Spectrometer was used. Samples were separated via a Thermo Scientific Vanquish Horizons UHPLC System functioning in binary mode.

Samples, which ranged from 40 to 150 mg, were collected into 13 × 100 mm borosilicate tubes with a Teflon-lined cap (catalog #60,827–453, VWR, West Chester, PA). Internal standards (10 μl) were added to samples and then lipids were extracted by the method of Bligh and Dyer^63^. Specifically, tissues were homogenized in 3 ml of methanol using a standard tissue homogenizer (Omni Int., Atlanta, USA) and then water and chloroform were added to a final ratio of 1:0.5:0.1 methanol:chloroform:water. Samples were sonicated for 30 s, capped, and incubated overnight at 48 °C. Samples were centrifuged to pellet insoluble materials (5 min at 5000 × g). The extract was reduced to dryness using a Speed Vac. The dried residue was reconstituted in 0.2 ml of the starting mobile phase solvent for untargeted analysis, sonicated for 15 s, then centrifuged for 5 min in a tabletop centrifuge before transfer of the clear supernatant to the autoinjector vial for analysis.

The lipids were separated by reverse phase LC using a Thermo Scientific Accucore Vanquish C18 + 2.1 (i.d.) × 150 mm column with 1.5 µm particles. The UHPLC used a binary solvent system at a flow rate of 0.26 mL/min with a column oven set to 55 °C. Prior to injection of the sample, the column was equilibrated for 2 min with a solvent mixture of 99% Mobile phase A1 (CH_3_CN/H_2_O, 50/50, v/v, with 5 mM ammonium formate and 0.1% formic acid) and 1% Mobile phase B1 (CH_3_CHOHCH_3_/CH_3_CN/H_2_O, 88/10/2, v/v/v, with 5 mM ammonium formate and 0.1% formic acid). After sample injection (typically 10 μL), the A1/B1 ratio was maintained at 99/1 for 1.0 min, followed by a linear gradient to 35% B1 over 2.0 min, then a linear gradient to 60% B1 over 6 min, followed by a linear gradient to 100% B1 over 11 min., which held at 100% B1 for 5 min, followed by a 2.0 min gradient return to 99/1 A1/B1. The column was re-equilibrated with 99:1 A1/B1 for 2.0 min before the next run. Each sample was injected two times for analysis in both positive and negative modes. For initial full scan MS (range 300–2000 m*/z*) the resolution was set to 120,000 with a data-dependent MS^2^ triggered for any analyte reaching a signal of 3e6 or above. Data-dependent MS^2^ were collected at 30,000 resolution.

Lipids were identified using Thermo Scientific’s Lipid Search 4.2 software. All lipids were identified by retention time, mass, and comparison to their expected MS^2^ fragmentation. The retention time interval was set to 0.01 min with precursor and product tolerances set to 5 ppm and 8 ppm respectively. Theoretical fragmentation was based on the LipidSearch higher energy C trap dissociation database developed specifically for the Q-Exactive. Transitions for all available mammalian lipid classes as well as transitions for the stable isotope internal standards were included for initial characterization.

After all lipid species were identified within a sample, the data in both positive and negative modes were aligned prior to statistical analyses. To reduce potential misidentified species, the m-Score threshold was set to 5.0 and the software ID quality filter was limited to A and B confidence scores. Peak retention time interval was raised to 0.05 min for alignment to account for potential peak drift. Compounds detected in both positive and negative mode were filtered by the mode in which ionization (and thus, signal) was most efficient. Only one signal (positive or negative) was reported. Signals for each internal standard were used to normalize relative recovery for each lipid class across samples. Signals were reported per mg tissue. Because of potential differences in ionization/fragmentation efficiency among different lipid species within a given lipid class, lipids are reported as relative across samples rather than as mass values.

### Statistical analysis

Statistical comparisons were made using analysis of variance (ANOVA). To correct for multiple comparisons we performed post hoc Tukey’s multiple comparison analysis. For individual lipid species, multiple comparisons for controlled for by controlling the False Discovery Rate (FDR) using Benjamini and Hochberg. Statistical significance was defined as adjusted p value of less than 0.05. Data are presented as mean ± standard error of the mean. Fold changes of ≥ 1.5 were considered significant. Statistical analysis including Pearson correlation coefficients were calculated using GraphPad Prism software.

### Supplementary Information

Supplementary Information.

## Data Availability

All data are contained within the manuscript. Data requests can be made to Brian Finck, Washington University School of Medicine, at bfinck@wustl.edu.
