# Supplementary material for: Dynamic changes in the mouse hepatic lipidome following warm ischemia reperfusion injury
Source: Sci Rep. 2024 Feb 13;14:3584. doi: 10.1038/s41598-024-54122-9 (PMC10864394; doi:10.1038/s41598-024-54122-9)

Supplemental Table 1: Lipids species significantly increased or decreased relative to sham.

Non-steatotic, 6 h		Steatotic, 6 h	
**Increased**	**Decreased**	**Increased**	**Decreased**
AcCa(18:2)	Co(Q9)	AcCa(18:0)	CL(18:2_16:1_16:1_20:3)
AcCa(18:0)	LPC(20:4)	AcCa(20:0)	CL(18:2_16:1_18:2_18:1)
DG(16:1_18:1)	LPC(22:6)	AcCa(24:1)	CL(18:2_18:2_18:2_18:2)
	PC(16:1_22:6)	PA(18:0_20:4)	CL(18:2_18:2_18:2_18:1)
	PC(18:2_20:4)		Co(Q9)
	PC(18:0_20:3)		Hex1Cer(d18:1_24:0)
	PE(16:0_20:4)		LPC(16:0)
			LPC(18:2)
			LPC(18:0)
			LPC(20:4)
			LPC(22:6)
			LPE(18:0)
			LPE(22:6)
			LPI(20:4)
			PC(16:0_20:5)
			PC(18:2_18:2)
			PC(16:1_22:6)
			PC(18:0_20:3)
			PC(18:0_22:6)
			PE(16:0_20:4)
			PE(18:1_18:2)
			PE(18:1_18:1)
			PE(18:0_18:1)
			PE(16:0_22:6)
			PE(18:0_20:4)
			PE(18:1_22:6)
			PE(18:0_22:6)
			PI(18:0_20:4)
			PS(16:0_22:6)
			PS(18:0_22:6)

Non-steatotic, 24 hr		Steatotic, 24 hr	
**Increased**	**Decreased**	**Increased**	**Decreased**
AcCa(18:2)	Co(Q9)	AcCa(18:0)	CL(18:2_16:1_16:1_20:3)
AcCa(18:0)	LPC(18:2)	DG(18:1_18:1)	CL(18:2_16:1_18:2_18:1)
AcCa(20:4)	LPC(20:4)		CL(18:2_18:2_18:2_18:2)
DG(16:1_18:2)	PC(16:1_22:6)		CL(18:2_18:2_18:2_18:1)
DG(16:1_18:1)	PC(18:2_20:4)		Cer(d18:1_24:0)
DG(18:2_18:2)	PC(18:0_20:3)		Co(Q9)
DG(18:1_18:2)	PE(16:0_20:4)		Hex1Cer(d18:1_24:0)
DG(16:0_22:6)	PS(16:0_22:6)		LPC(16:1)
PC(16:0_18:2)			LPC(16:0)
			LPC(18:2)
			LPC(18:1)
			LPC(18:0)
			LPC(20:4)

			LPC(22:6)<br>PC(16:0_20:5)<br>PC(18:2_18:2)<br>PC(18:1_18:2)<br>PC(18:1_18:1)<br>PC(18:0_18:1)<br>PC(16:1_22:6)<br>PC(18:1_20:3)<br>PC(18:0_20:3)<br>PE(16:0_20:4)<br>PE(18:1_18:1)<br>PE(18:0_18:1)<br>PE(18:0_20:4)<br>PE(18:1_22:6)<br>PI(18:1_20:4)<br>PS(16:0_20:4)<br>PS(16:0_22:6)<br>PS(18:0_22:6)

**Non-steatotic, 72 hr**		**Steatotic, 72 hr**	
**Increased**	**Decreased**	**Increased**	**Decreased**
AcCa(18:0)<br>AcCa(20:4)<br>Hex1Cer(d18:1_24:0)<br>PI(18:0_18:1)	DG(16:1_18:1)<br>DG(18:1_18:2)<br>LPC(18:2)<br>PC(18:2_18:2)<br>PC(16:1_22:6)		AEA(16:0)<br>AcCa(18:2)<br>AcCa(20:4)<br>CL(18:2_16:1_16:1_20:3)<br>CL(18:2_16:1_18:2_18:1)<br>CL(18:2_18:1_16:1_18:1)<br>CL(18:2_18:2_18:2_18:2)<br>CL(18:2_18:2_18:2_18:1)<br>Cer(d18:1_24:0)<br>Co(Q9)<br>DG(16:1_18:1)<br>DG(18:1_18:2)<br>Hex1Cer(d18:1_24:0)<br>LPC(16:1)<br>LPC(16:0)<br>LPC(18:2)<br>LPC(18:1)<br>LPC(18:0)<br>LPC(20:4)<br>LPC(22:6)<br>LPE(18:1)<br>LPE(18:0)<br>LPE(22:6)<br>LPI(20:4)<br>PC(16:1_16:1)<br>PC(16:0_16:1)<br>PC(16:1_18:1)<br>PC(16:0_18:1)<br>PC(16:0_20:5)<br>PC(18:2_18:2)<br>PC(16:0_20:4)<br>PC(18:1_18:2)

			PC(18:1_18:1)<br>PC(18:0_18:1)<br>PC(16:1_22:6)<br>PC(18:2_20:4)<br>PC(16:0_22:6)<br>PC(18:1_20:3)<br>PC(18:0_20:3)<br>PC(18:0_22:6)<br>PE(16:0_20:4)<br>PE(18:1_18:2)<br>PE(18:1_18:1)<br>PE(18:0_18:1)<br>PE(16:0_22:6)<br>PE(18:0_20:4)<br>PE(18:1_22:6)<br>PE(18:0_22:6)<br>PI(18:0_18:1)<br>PI(18:1_20:4)<br>PS(16:0_20:4)<br>PS(16:0_22:6)<br>PS(18:0_20:4)<br>PS(18:0_22:6)

Supplemental Table 2: Lipid species with significant positive or negative correlation with plasma ALT.

Non-steatotic, 6 hr		Steatotic, 6	
**Positive**	**Negative**	**Positive**	**Negative**
PC(16:0_16:1)<br>PC(16:0_18:1)<br>PC(17:0_18:1)<br>AcCa(22:0)<br>AcCa(24:1)	LPC(18:3)<br>LPC(18:2)<br>LPC(20:4)<br>LPC(22:6)<br>PC(14:0_20:4)<br>PC(15:0_20:4)<br>PC(17:0_18:2)<br>PC(16:1_20:5)<br>PC(18:3_18:2)<br>PC(16:0_20:5)<br>PC(18:2_18:2)<br>PC(16:0_20:4)<br>PC(17:0_20:4)<br>PC(19:0_18:2)<br>PC(16:1_22:6)<br>PC(18:2_20:4)<br>PC(16:0_22:6)<br>PC(18:1_20:4)<br>PC(18:1_20:3)<br>PC(20:0_18:2)<br>PC(18:0_22:6)<br>PC(20:0_20:4)<br>PC(20:0_20:3)<br>PC(20:4_22:6)<br>PC(20:1_22:6)<br>PC(20:0_22:6)<br>DG(18:3_18:2)<br>DG(18:2_18:2)<br>DG(18:2_20:4)<br>DG(16:0_22:6)<br>DG(18:0_20:4)<br>DG(18:2_22:6)<br>DG(22:5_18:2)<br>DG(18:1_22:5)<br>PS(16:0_20:5)<br>PS(16:0_20:4)<br>PS(18:0_18:2)<br>PS(16:0_22:6)<br>PS(18:0_20:5)<br>PS(18:0_20:4)<br>PS(20:4_20:4)<br>PS(18:1_22:6)<br>PS(18:0_22:6)<br>PS(20:4_22:6)<br>CL(18:2_16:1_18:2_18:1)<br>CL(18:2_18:1_16:1_18:1)<br>CL(18:3_18:2_18:2_18:2)	PC(18:0_16:0)<br>DG(16:1_18:2)<br>AcCa(22:1)<br>AcCa(24:1)	LPC(16:1)<br>LPC(18:3)<br>LPC(18:2)<br>LPC(18:1)<br>LPC(18:0)<br>LPC(20:5)<br>LPC(20:4)<br>LPC(22:6)<br>PC(16:1_18:3)<br>PC(14:0_20:4)<br>PC(15:0_20:4)<br>PC(16:1_20:5)<br>PC(18:1_18:2)<br>PC(16:1_22:6)<br>PC(20:0_20:3)<br>PC(20:4_22:6)<br>PS(16:0_20:5)<br>PS(16:0_20:4)<br>PS(18:0_20:5)<br>PS(18:0_20:4)<br>CL(18:2_16:1_16:1_20:3)<br>CL(18:2_16:1_18:2_18:1)<br>CL(18:2_18:1_16:1_18:1)<br>CL(18:3_18:2_18:2_18:2)<br>CL(18:2_18:2_18:2_18:2)<br>CL(18:2_18:2_18:2_18:1)<br>CL(18:2_18:2_18:2_22:6)<br>Hex1Cer(d18:1_22:0)<br>Hex1Cer(d18:1_24:0)<br>Co(Q8)<br>Co(Q9)<br>Co(Q10)<br>PG(22:6_22:6)<br>LPI(18:1)<br>LPI(18:0)<br>LPI(20:4)<br>LPI(20:3)<br>PI(16:0_18:2)<br>PI(16:0_20:5)<br>PI(16:0_20:4)<br>PI(18:1_18:1)<br>PI(17:0_20:4)<br>PI(18:1_20:4)<br>PI(18:0_20:4)<br>PI(19:0_20:4)

	CL(18:2_18:2_18:2_18:2)<br>CL(18:2_18:2_18:2_18:1)<br>CL(18:2_18:2_18:2_22:6)<br>Hex1Cer(d18:1_22:0)<br>Hex1Cer(d18:1_23:0)<br>Hex1Cer(d18:1_24:0)<br>AcCa(18:2)<br>AcCa(20:4)<br>Co(Q8)<br>Co(Q9)<br>Co(Q10)<br>PG(22:6_22:6)<br>LPI(18:0)<br>LPI(20:4)<br>PI(16:1_18:2)<br>PI(16:0_18:2)<br>PI(16:0_20:4)<br>PI(18:1_18:1)<br>PI(18:0_18:1)<br>PI(17:0_20:4)<br>PI(18:2_20:4)<br>PI(16:0_22:6)<br>PI(18:0_20:5)<br>PI(18:1_20:4)<br>PI(18:0_20:4)<br>PI(19:0_20:4)<br>PI(20:4_20:4)<br>PI(18:0_22:6)<br>PI(18:0_22:4)<br>PI(20:0_20:4)<br>PE(16:0p_20:4)<br>PE(18:0p_18:2)<br>PE(18:3_18:2)<br>PE(16:0_20:5)<br>PE(18:2_18:2)<br>PE(16:0_20:4)<br>PE(18:1_18:2)<br>PE(18:1_18:1)<br>PE(18:0_18:1)<br>PE(16:0p_22:6)<br>PE(15:0_22:6)<br>PE(16:0p_22:5)<br>PE(18:0p_20:4)<br>PE(16:1_22:6)<br>PE(16:0_22:6)<br>PE(18:1_20:4)<br>PE(18:0_20:4)<br>PE(20:0_18:2)<br>PE(18:1e_22:6)<br>PE(18:0p_22:6)<br>PE(18:0p_22:5)<br>PE(19:0_20:4)<br>PE(18:3_22:6)		PI(18:0_22:6)<br>PI(18:0_22:5)<br>PI(20:0_20:4)<br>PE(16:1_20:4)<br>PE(18:0p_22:5)<br>PE(18:3_22:6)

	PE(18:2_22:6)<br>PE(18:1_22:6)<br>PE(18:0_22:6)<br>PE(20:0_22:6)		

**Non-steatotic, 24 hr**		**Steatotic, 24 hr**	
**Positive**	**Negative**	**Positive**	**Negative**
PC(16:0_18:1)<br>AcCa(18:2)<br>AcCa(20:0)<br>AcCa(22:0)<br>AcCa(24:1)<br>PI(18:1_18:1)<br>PE(16:0_16:0)	PC(18:3_18:2)<br>PC(17:0_20:4)<br>PC(19:0_18:2)<br>CL(18:2_16:1_16:1_20:3)<br>LPE(18:0)<br>LPE(22:6)<br>PI(16:0_20:4)<br>PE(16:1_18:2)<br>PE(18:2_18:2)<br>PE(18:0_18:1)<br>PE(16:1_22:6)<br>PE(18:0_20:4)<br>PE(18:2_22:6)<br>PE(18:1_22:6)<br>PE(18:0_22:6)	AcCa(22:1)	PC(16:0_20:5)<br>DG(18:1_18:1)

**Non-steatotic, 72 hr**		**Steatotic, 72 hr**	
**Positive**	**Negative**	**Positive**	**Negative**
	PC(18:2_18:2)<br>PS(16:0_20:4)	DG(18:1_18:2)<br>DG(18:1_22:5)	LPC(20:2)<br>PC(16:1_18:1)<br>PC(17:1_18:2)<br>PC(17:0_18:2)<br>PC(17:0_18:1)<br>DG(18:1_18:2)<br>DG(18:1_22:5)<br>PS(18:0_18:2)<br>Co(Q8)

Supplemental Figure 1. Body weight and liver histology of mice fed a chow or 42% HF diet. **A**. Body weight on non-steatotic or steatotic diet. **B**. Representative H&E liver sections. **C**. Intrahepatic triglyceride content. *p<0.05 non-steatotic vs steatotic diet at specified reperfusion time point. #p<0.05 non-steatotic vs non-steatotic sham. Values are mean ± SEM. Statistical comparisons were made using ANOVA with post-hoc Tukey. Statistical significance was defined as p<0.05. * indicates p<0.05 between non-steatotic and steatotic, # indicates p<0.05 between sham non-steatotic and non-steatotic IR at specified reperfusion time point. ϕ indicates p<0.05 between sham steatotic and steatotic IR at specified reperfusion time point.

Supplemental Figure 1.

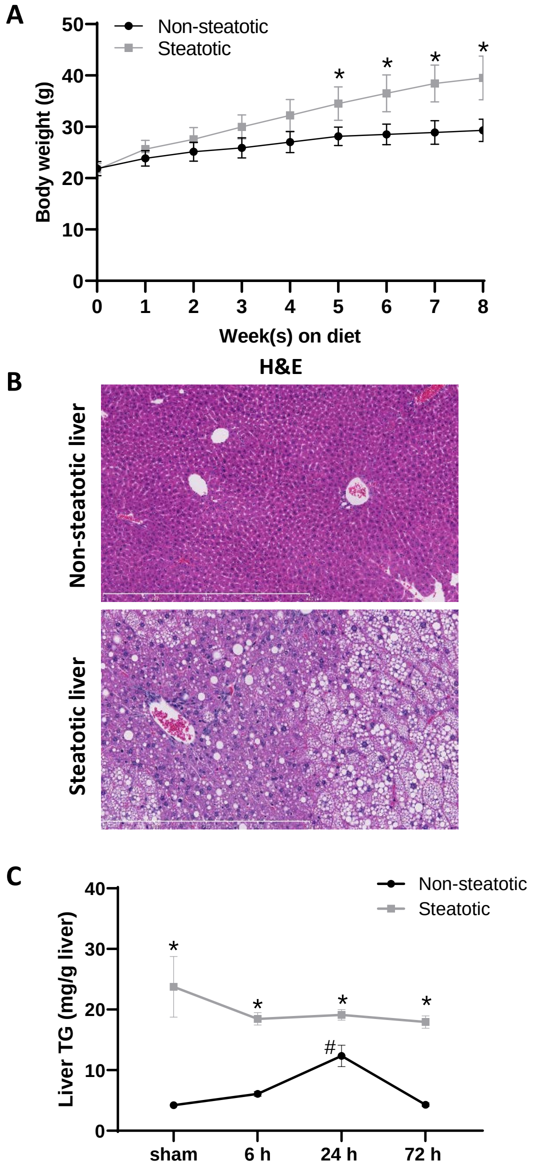

Supplemental Figure 2. PA content following sham or IR surgery. Statistical comparisons were made using ANOVA with post-hoc Tukey. Statistical significance was defined as $p<0.05$. * indicates $p<0.05$ between non-steatotic and steatotic, # indicates $p<0.05$ between sham non-steatotic and non-steatotic IR at specified reperfusion time point. ϕ indicates $p<0.05$ between sham steatotic and steatotic IR at specified reperfusion time point. Values are mean ± SEM. n = 4 per sham group, 8-10 per IR surgery group. Sham indicates sham surgery. 6 h, 24 h, 72 h indicates hours of reperfusion following IR surgery. Black bars are chow fed mice and represent non-steatotic liver. Gray bars are 42% HF fed mice and represent steatotic liver.

Supplemental Figure 2.

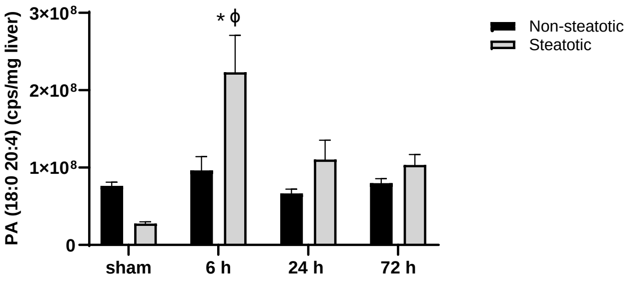

Supplement: Supplementary file 1 — Supplementary Information. [file 41598_2024_54122_MOESM1_ESM.pdf]
